# Using sediment grain size characteristics to assess effectiveness of mechanical sand barriers in reducing erosion

**DOI:** 10.1038/s41598-020-71053-3

**Published:** 2020-08-19

**Authors:** Yunhu Xie, Xiaohong Dang, Yujuan Zhou, Zhihui Hou, Xiaojia Li, Hongtao Jiang, Dandan Zhou, Ji Wang, Chunxing Hai, Ruiping Zhou

**Affiliations:** 1 College of Geographical Science, Inner Mongolia Normal University, Hohhot, 010022 China; 2 College of Desert Control Science and Engineering, Inner Mongolia Agricultural University, Hohhot, 010018 China; 3 Inner Mongolia Water Resources and Hydropower Survey and Design Institute, Huhhot, 010020 China; 4 Institute of Rural Economic and Information, Inner Mongolia Academy of Agricultural & Animal Husbandry Sciences, Hohhot, 010031 China

**Keywords:** Environmental sciences, Environmental impact

## Abstract

Wind and sand control features are important tools for limiting desertification. Sand barriers are one of the oldest engineering measures used to reduce wind-sand hazards. Their efficacy and exact mechanism by which they work has remained a topic of scientific debate however. Sediment grain-size distributions can help constrain their utility and function. This research analyzed sediment grain size distributions in samples collected from areas around six different types of sand barriers installed along the southeastern margin of the Tengger Desert. Results were compared with sediment from a bare dune area (no barriers) used as a control. The barrier area samples contained high proportions of coarse sand and relatively low proportions of silty sand and very fine sand. Fine and medium sand were present but clay was not. The lower proportions of fine sand and higher proportions of coarse sand relative to bare dunes documented an effective reduction in aeolian transport by the barriers. Samples from the barrier areas also showed poorer sorting relative to bare dune areas. This appeared as lower kurtosis values and wider frequency distribution curves relative to those measured from bare dunes samples. The wider cumulative frequency curves for samples from the barrier areas likely reflects the higher proportion of coarse-grained material. The Straw/1.5 and PLA/1 barrier types hosted greater sediment accumulation than that observed for the other barrier types (Straw/1, PLA/1.5, Mixed/1 and Mixed/1.5). Sediment grain size distributions showed that the base and middle slope areas of the dune experienced deposition, while the top of the dunes experienced erosion. The Straw/1 barrier (straw installed as a 1 × 1 m grid) performed best in terms of installation costs and protective effects for the study area. This study demonstrates how sediment grain size distributions can be used as quantitative proxies for sand barrier performance in reducing desertification.

## Introduction

Desertification is a major environmental problem that disrupts industrial activity, agriculture, transportation, mining and residential life. Economic and social impacts have led to various measures aimed at preventing desertification^1–4^. Mechanical sand barriers typically used to combat desertification^5–7^ can effectively control the movement of sand particles by increasing the surface aerodynamic roughness and by reducing wind speed near the surface. Mechanical sand barriers disrupt wind-sand flow and also reduce the ability of wind to carry sand leading to reduced erosion and transport and greater accumulation in different areas. Deposition of fine particles through this process leads to higher proportions of fine grained sediment within and around the sand barrier, which in turn supports soil development and vegetation^8^. Grain-size distributions in surface sediments can therefore serve as a spatial proxy for aeolian processes. Underlying surface factors can also influence wind erosion^9,10^, which represents a first order driving mechanism in desertification. As the major physical property of sediment, measures of sediment grain-size include size (diameter) and various proportional metrics of sand particle size classes. Grain-size distributions depend on factors such as transport medium, transport mode, sedimentary environment and climate. Aeolian velocity thresholds for erosion, transport and deposition also depend on sediment grain size. Grain size distributions can thereby record evolution of the sedimentary environment and this parameter has informed numerous studies on desertification^11,12^. Commonly used grain size parameters include mean particle size, standard deviation and kurtosis^11^. Particle frequency distributions, probability accumulation distributions and source discriminant functions have also been used to interpret sedimentary environments^12,13^.

Recent, adjacent studies of the Tengger desert have interpreted wind-sand flow, particle size distributions and the effectiveness of protective measures. Mechanical sand barriers cause a blocking effect that increases the percentage of medium to coarse sand and decreases the percentage of fine sand to cause an overall coarsening of surface sediments^14–16^. Mechanical sand barriers can intercept more than 90% of the sand. Particle size ranges of 1.32Φ to 4Φ are prone to wind-sand hazards^17^. Following sand barrier installation, the mean particle size inside the sand barrier increases and sediment sorting became poorer. However, skewness and kurtosis did not vary significantly^18^. Consistent maintenance of the sand barrier and sand surface stabilization supports long term soil development^2^.

The Tengger Desert is the fourth largest desert in the world. It has exhibited a tendency of continuous expansion over the last century. Aeolian desertification, which refers to the wind-induced loss of otherwise vegetated land, occurs along shifting marginal areas of the desert. Mechanical sand barriers installed along marginal areas facing the prevailing wind direction can effectively prevent aeolian desertification. This research compared changes in surface sediment (0–5 cm) that accompanied installation of six different types of mechanical sand barriers. These barriers were installed along the southeastern margin of the Tengger Desert. Nearby bare dunes served as a control area for determining the effects of different sand barriers types on sediment grain size distributions as they relate to the different sedimentary processes of erosion, transport and deposition. This research specifically sought to (1) study the effects of three different types of mechanical sand barriers on surface sediment grain size distributions, (2) quantify variation in wind-sand flow and spatial distribution of sediment around sand barriers and (3) determine the protective efficiency of different types of sand barrier for large scale mitigation efforts. To meet these objectives, the study describes sediment grain-size distributions and uses them to interpret wind-sand flow parameters around the different types of sand barrier. The study also used sedimentary parameters to compare barrier types in terms of their effectiveness in controlling large-scale erosional processes. This research can inform mitigation efforts in arid environments experiencing desertification and in coastal areas under developmental stress.

### Site description

**Figure 1:**
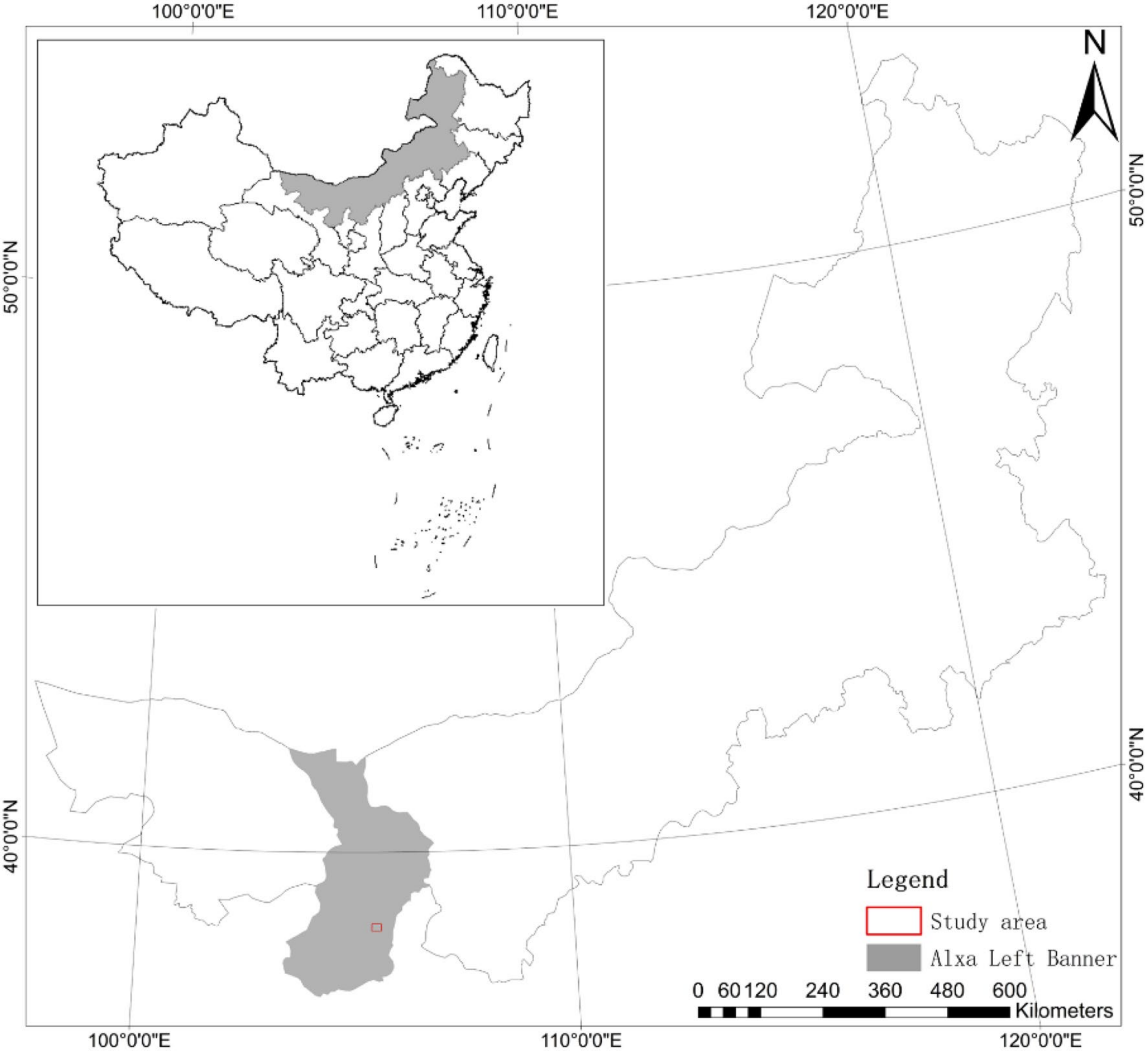
Map showing the location of the study area in Alxa Left Banner, China.

The research area is located in Alxa Left Banner of Inner Mongolia along the southeastern margin of the Tengger Desert (38°43′33″N,105°31′27″E; altitude approx.1370 m) (Fig. 1). This region has a dry climate and experiences severe long term loss of vegetation. Strong wind erosion causes different desert landscape types including barchan dunes and dune chains, knap-shaped dunes, network dunes, sabkhas and wind erosion depressions. The area experiences high evaporation rates, limited rainfall, high summer temperatures, cold winter temperatures, large day-night temperature differences and strong sandstorms. Temperature profiles resemble those predicted for a continental arid climate at this latitude. Annual mean temperature is 7.7 °C annual precipitation is 210 mm and mainly falls in July, August and September. Annual evaporation is 2,362.7 mm, which exceeds annual precipitation by an order of magnitude. Average wind velocity is 3.1 m/s, while instantaneous wind velocity is 17.9 m/s. The land itself consists of sandy soil, while the zonal soil is gray desert soil. The area hosts simple vegetation regimes consisting mostly of single species. These include *Artemisia desertorum*, *Haloxylon ammodendron*, H*hedysarum scoparium* and *Agriophyllum squarrosum*.

## Materials and methods

### Experimental material

**Figure 2:**
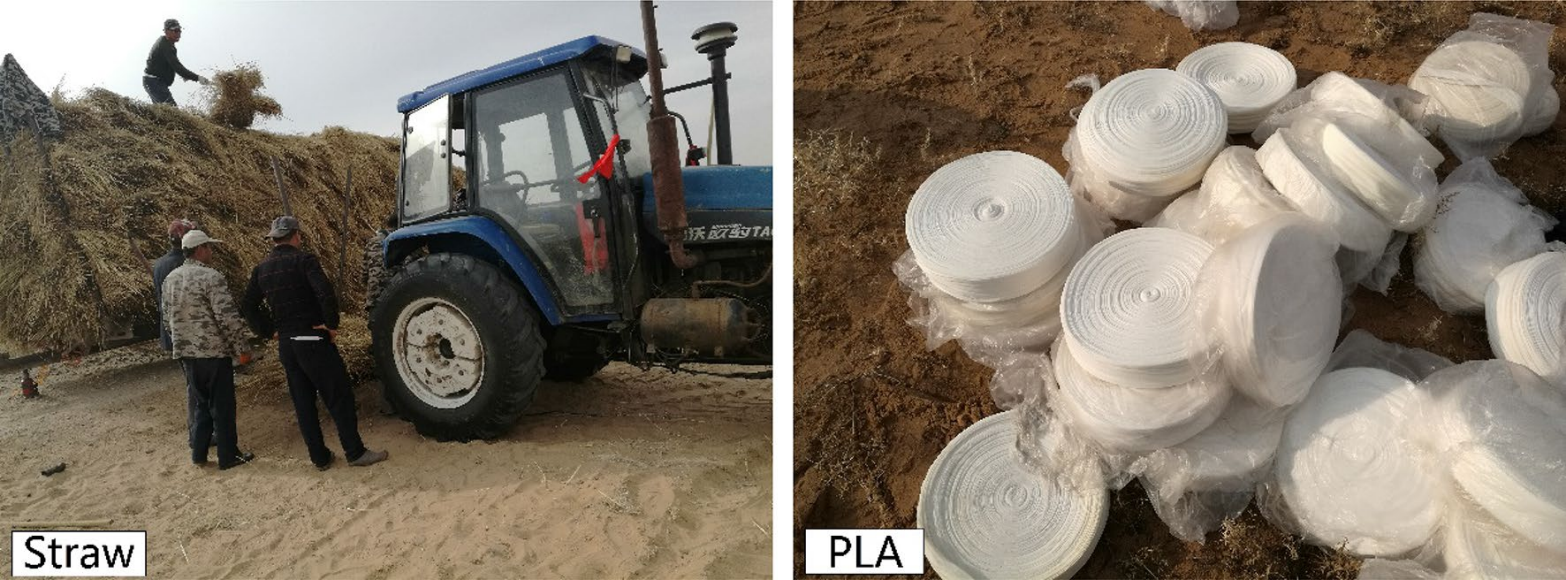
Sand barrier materials including wheat straw (left) and PLA tubing (right).

**Figure 3:**
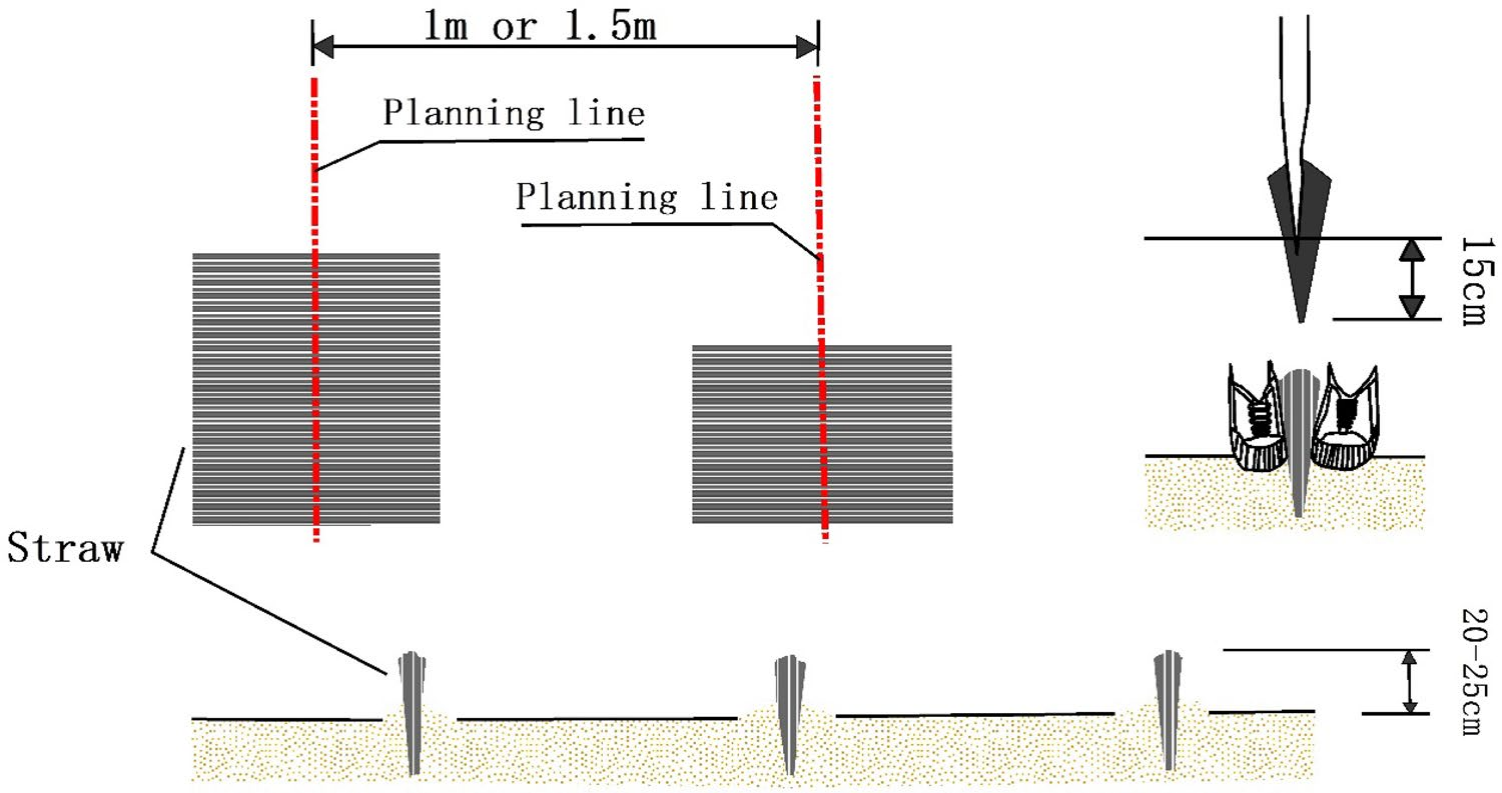
Installation of straw barrier with 1 m and 1.5 m grid dimensions.

**Figure 4:**
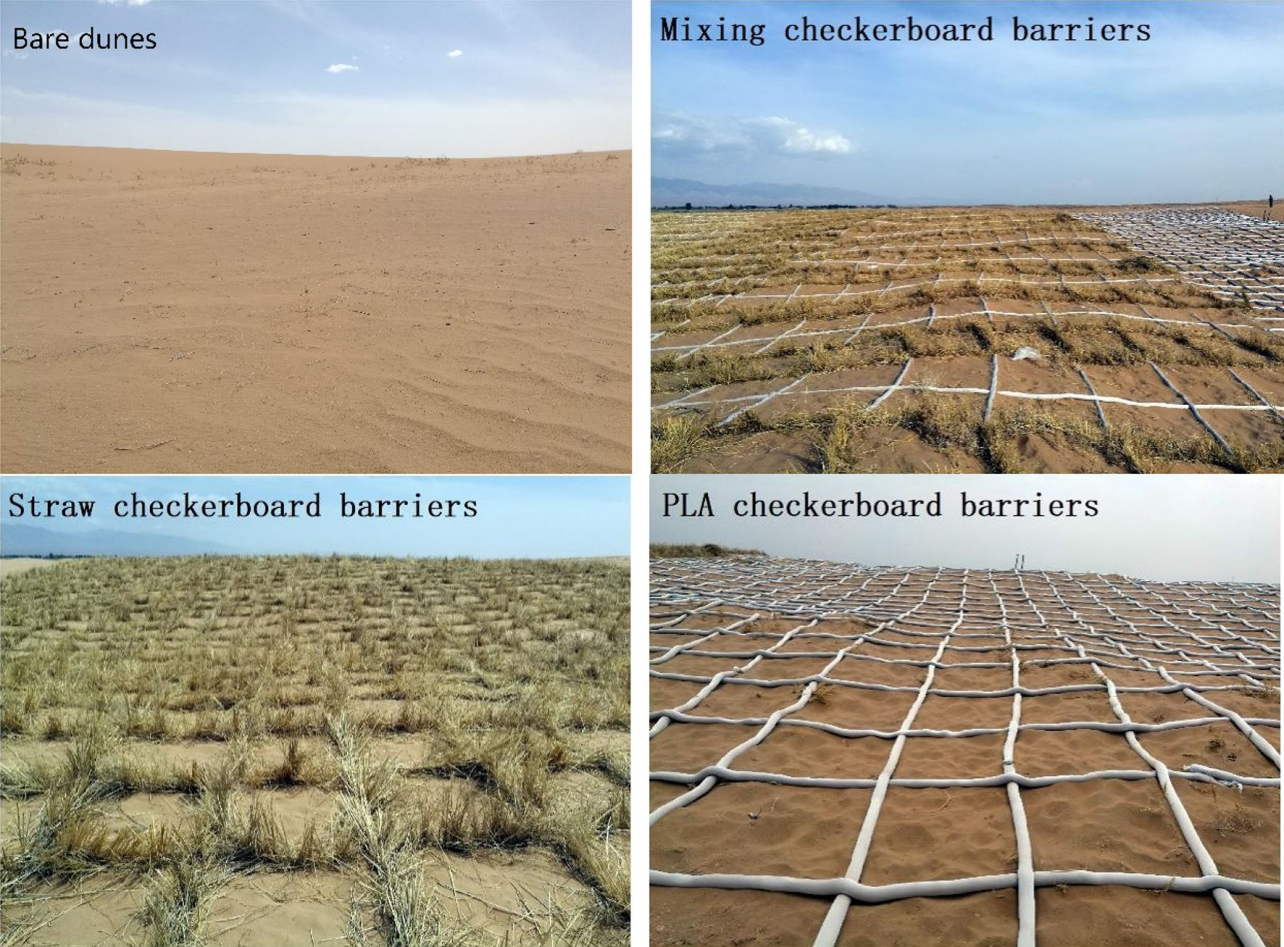
Bare sand dunes (upper left) and three types of mechanical sand barrier: mixed barrier (upper right), straw checkerboard barrier (lower left) and PLA checkerboard barrier (lower right).

The sand barrier materials used in this study included wheat straw and Poly Lactic Acid (PLA; a bioplastic product) tubing (Fig. 2). Sections of wheat straw extends about 75 cm in length. These have the advantages of being low cost, having simple installation and being made of natural materials. The straw was implanted by shovel within the surface of the sand dunes following a gridded barrier pattern. Both sides of the straw are fixed with sand (Fig. 3). PLA is a light bioplastic that biodegrades without leaving harmful residuals in the environment. PLA tubing can reach 500 m and can be filled to 10 cm diameter with local sand. This material can be installed in a gridded pattern established and optimized by previous studies (Fig. 4).

### Site description

This study analyzed an area of six sand dunes about 25 km south of Bayanhaote in May 2017. Bayanhaote serves as the local government seat for Alxa Left Banner. The sand dunes exhibited gentle slopes and had no vegetative cover. Sand barriers were installed on the dunes so as to cover lower, middle and upper parts of the dune. The sand barriers were categorized according to the material and spacing as shown above (Fig. 2). Straw/1 and Straw/1.5 used only straw materials installed as 1 × 1 m and 1.5 × 1.5 m grids, respectively. PLA/1 and PLA/1.5 used only PLA tubing installed as 1 × 1 m and 1.5 × 1.5 m grids, respectively, Mixed/1 and Mixed/1.5 used both materials with the 1 × 1 m and 1.5 × 1.5 m grid patterns. The straw used in the straw and mixed barriers reached 20–25 cm height and the filled PLA tubing reached 10 cm height. The mixed barriers ranged from 10 to 25 cm height (Table 1). Sand barriers were installed from the base to the top of the dune slope for maximum impact.

**Table 1:** Sand barrier type and specifications.

Material type	Checkerboard grid size (m)	Label	Sand barrier area (m^2^)	Barrier height (cm)	Barrier width (cm)
Straw checkerboard barriers	1 × 1	Straw/1	12,666.73	20–25	5–8
Straw checkerboard barriers	1.5 × 1.5	Straw/1.5	14,000.07	20–25	5–8
PLA checkerboard barriers	1 × 1	PLA/1	9,333.38	10	10
PLA checkerboard barriers	1.5 × 1.5	PLA/1.5	8,000.04	10	10
Mixed barriers	1 × 1	Mixed/1	10,000.05	10–25	5–10
Mixed barriers	1.5 × 1.5	Mixed/1.5	12,666.73	10–25	5–10

### Sample collection

**Figure 5:**
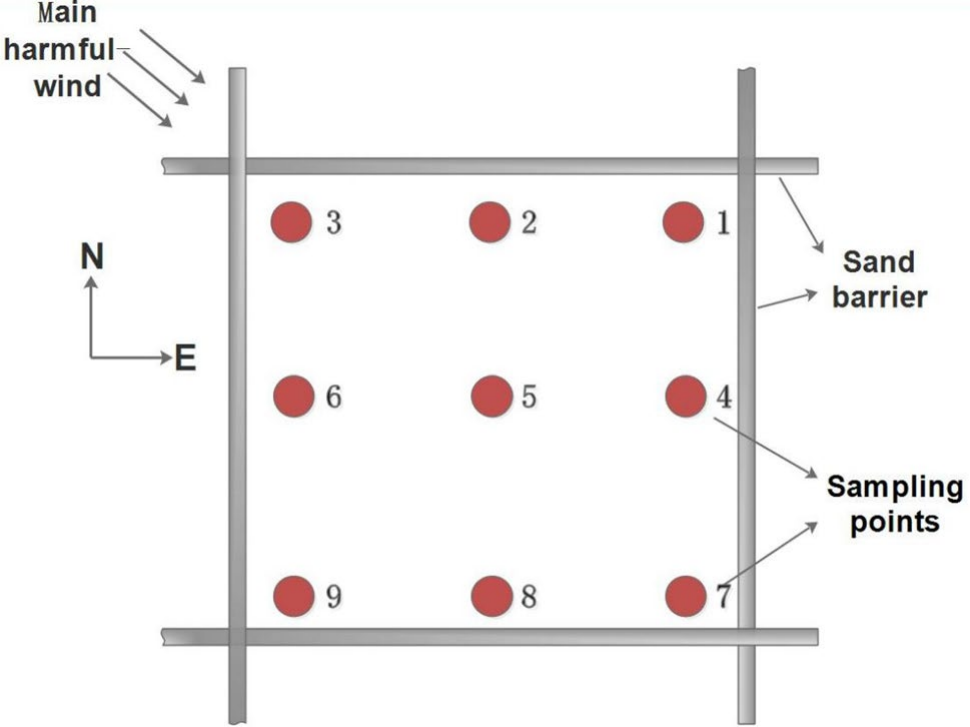
Schematic diagram of sampling points.

Sediment samples were collected from the six sand dunes with sand barriers. Dune areas were parsed according to different slope positions (base, middle and top) and the square areas within the barrier grids were parsed into 9 sample points (Fig. 5). Samples of 150 g were collected from the upper 0–5 cm of the sediment surface. Samples were transferred to numbered, self-sealing bags and tagged according to their recorded location. Sediment samples were collected from the bare dunes area at the same time as control material for each slope position. All samples were dried and further processed at the lab after sampling.

### Determination of sediment grain size

Sample preparation and grain size measurements were performed at the Key Laboratory of Mongolian Plateau Environment & Global Change. Sample preparation included air drying, sieving for impurities, removal of organic matter and desalting^19^. The quarter method was used to select 5 g of sample for analysis using a Master Sizer 3000 laser particle size analyzer produced by Malvern, UK. The instrument included a Hydro LV type large-capacity sampler which handles large and highly variable samples for which measurement ranges can span 0.01 to 3,500 μm with precision of 0.6%. Replicate measurement samples varied by less than 0.5%, and reproducibility was less than 1%. Every soil sample was analyzed three times. Reporting used United States Department of Agriculture (USDA) sediment grain size classification standards which include gravel (> 2000 μm), very coarse sand (1,000–2000 μm), coarse sand (500–1,000 μm), medium sand (250–500 μm), fine sand (100–250 μm), very fine sand (50–100 μm), silty sand (2–50 μm) and clay (< 2 μm)^20^. Excel 2010, SPSS 23.0 and Origin2017 were used to analyze the grain size data.

### Grain-size characteristics

Standard grain size parameters were estimated using Udden-Wentworth grain size criteria and then transformed using Kumdein's algorithm to calculate Φ^21^. This conversion is as follows:

1 $$ \Phi = - \log_{2} D $$

Grain-size characteristics were calculated using the Folk-Ward graphic method^13^ which uses average grain size of sediments ($M_{Z}$), sorting (${\upsigma }$), skewness ($S_{K}$) and kurtosis ($K_{g}$). The calculations were as follows:

2 $$ M_{Z} = \frac{{\left( {\Phi_{16} + \Phi_{50} + \Phi_{84} } \right)}}{3} $$

3 $$ \sigma = \frac{{\Phi_{25} - \Phi_{16} }}{4} + \frac{{\Phi_{95} - \Phi_{5} }}{6.6} $$

The smaller the value of ${\upsigma }$, the smaller the degree of size dispersion within the sediment sample. The more concentrated the distribution, the better the sorting. Estimates of generally fell within seven categories (Table 2).

4 $$ S_{K} = \frac{{\Phi_{16} + \Phi_{84} - 2\Phi_{50} }}{{2\left( {\Phi_{84} - \Phi_{16} } \right)}} + \frac{{\Phi_{5} + \Phi_{95} - 2\Phi_{50} }}{{2\left( {\Phi_{95} - \Phi_{5} } \right)}} $$

**Table 2:** Descriptive terms for sorting.

σ < 0.35	0.35 < σ ≤ 0.50	0.50 < σ ≤ 0.71	0.71 < σ ≤ 1.00	1.00 < σ ≤ 2.00	2.00 < σ ≤ 4.00	σ > 4.00
Extremely good	Good	Better	Medium	Worse	Bad	Worst

The skewness reflects size distribution characteristics. These fell into five categories (Table 3).

**Table 3:** Descriptive terms for the skewness.

− 1.0 ≤ SK < − 0.3	− 0.3 ≤ SK < − 0.1	− 0.1 ≤ SK < 0.1	0.1 ≤ SK < 0.3	0.3 ≤ SK < 1.0
Very negatively skewed	Negatively skewed	Symmetric	Fine skewed	Very fine skewed

The kurtosis coefficient (Kg) (Table 4) describes the degree of concentration of different sized particles relative to the average particle size. This calculation is as follows:

5 $$ K_{g} = \frac{{\Phi_{95} - \Phi_{5} }}{{2.44\left( {\Phi_{75} - \Phi_{25} } \right)}} $$

**Table 4:** Description of kurtosis.

$K_{g}$ ≤ 0.67	0.67 < $K_{g}$ ≤ 0.9	0.9 < $K_{g}$ ≤ 1.11	1.11 < $K_{g}$ ≤ 1.56	1.56 < $K_{g}$ ≤ 3.00	$K_{g}$ > 3.00
Very platykurtic	Platykurtic	Mesokurtic	Leptokurtic	Very leptokurtic	Extremely leptokurtic

### Mean distance between cumulative frequencies of sediment particle size

The mean distance between cumulative frequencies in sediment particle size (d) reflects variation in sedimentary processes contributing to different sample sites. It also constrains aeolian erosion of sediments^22^. The calculation is as follows:

6 $$ d = \sqrt {\left( {P - \overline{P}} \right)^{2} \left( {K - 1} \right)} $$

where d is the mean distance between cumulative frequencies of sediment grain size, P is the cumulative frequency of sediment grain size at sample sites, ${\overline{\text{P}}}$ is the average value of cumulative frequencies among seven sample sites and K-1 are the degrees of freedom (where K = 7).

## Results and analysis

### Sediment grain size distributions

Surface sediment samples collected from bare dunes consisted primarily of fine sand, medium sand and coarse sand. These samples showed higher relative proportions of medium sand, which varied from 39.3 to 59.34${\% }$. Fine and coarse sand proportions varied from 2.27 to 33.29% and 8.14 to 55.82%, respectively. Fine and silty sand content were less than 5%. Samples from along the middle of the slope exhibited higher proportions of fine sand (55.82%) than those from the base and top of the slope. Samples from the middle of the slope gave lower proportions of medium and coarse grained sand (39.30% and 29.27%, respectively) relative to that measured in samples from the base and top of the slope. Medium sand made up 55.22% and 55.34% of sediment in samples from the base and middle slope (Table 5).

**Table 5:** Sediment grain-size characteristics from bare dunes (control) area and six different types of mechanical sand barrier.

Types	Position	Clay (> 8φ)/%	Silty (4 ~ 8φ)/%	Very fine sand (3 ~ 4φ)/%	Fine sand (2 ~ 3φ)/%	Medium sand (1 ~ 2φ)/%	Coarse (< 1φ)/%
Bare dunes	Base of the slope	0.00	0.11 ± 0.43^BCFGa^	3.24 ± 1.28^ADa^	8.14 ± 4.35^Ba^	55.22 ± 5.36^Ba^	33.29 ± 5.26^CDEFa^
	Middle of the slope	0.00	0.09 ± 0.37^Abd^	2.07 ± 1.32^GIb^	55.82 ± 6.24^Ab^	39.30 ± 6.24^ACDb^	2.72 ± 3.34^AEb^
	Top of the slope	0.00	0.07 ± 0.32^AEHcd^	4.06 ± 1.43^Gc^	19.5 ± 7.31^Ec^	55.34 ± 8.32^BCEc^	21.03 ± 6.29^Gc^
Straw/1	Base of the slope	0.00	0.52 ± 0.59^ACab^	6.51 ± 2.97^Aabc^	39.87 ± 11.63^Aa^	40.04 ± 7.49^ACab^	13.06 ± 7.62^ACa^
	Middle of the slope	0.00	1.07 ± 1.01^Ba^	7.08 ± 4.18^Eb^	47.42 ± 11.56^Aa^	35.1 ± 11.09^Ab^	9.33 ± 4.51^ACEFa^
	Top of the slope	0.00	0.15 ± 0.19^Ab^	3.79 ± 1.77^ACDc^	36.75 ± 13.64^CDa^	45.2 ± 8.33^ADFa^	14.11 ± 7.11^CDa^
Straw /1.5	Base of the slope	0.00	0.65 ± 0.43^Aa^	6.74 ± 2.71^Aa^	48.63 ± 11.67^Aa^	33.52 ± 8.69^ACa^	10.47 ± 5.86^AFa^
	Middle of the slope	0.00	0.39 ± 0.57^Aa^	5.18 ± 2.72^ABDEIa^	49.71 ± 15.01^Aa^	35.89 ± 9.93^Aa^	8.82 ± 8.25^ Da^
	Top of the slope	0.00	0.45 ± 0.93^Aa^	4.94 ± 1.99^Aa^	29.15 ± 6.79^ACb^	47.27 ± 4.71^ADFb^	18.19 ± 4.26^ACEb^
PLA/1	Base of the slope	0.00	0.12 ± 0.30^BDEHa^	6.55 ± 1.68^Aa^	47.8 ± 6.14^Aa^	33.05 ± 4.44^Ca^	12.47 ± 3.01^AFa^
	Middle of the slope	0.00	0.07 ± 0.19^Aa^	4.02 ± 1.16^BDHIbc^	46.82 ± 12.73^ACa^	37.72 ± 8.42^AEab^	11.38 ± 6.30^FGa^
	Top of the slope	0.00	0.07 ± 0.22^AFIa^	2.88 ± 1.78^BDFc^	46.22 ± 15.40^DFa^	40.51 ± 9.15^Fb^	10.31 ± 8.25^DFHa^
PLA/1.5	Base of the slope	0.00	0.39 ± 0.53^AGHa^	4.9 ± 2.02^AEa^	39.75 ± 12.16^Aa^	40.7 ± 6.65^Aa^	14.27 ± 7.78^AFa^
	Middle of the slope	0.00	0.09 ± 0.26^Aab^	4.33 ± 1.73^BDHIa^	37.13 ± 8.70^BCa^	43.29 ± 6.15^BDEa^	15.17 ± 4.66^BGa^
	Top of the slope	0.00	0.01 ± 0.02^BCGHIb^	1.26 ± 1.67^BGb^	12.3 ± 10.70^BEb^	59.2 ± 7.29^Bb^	27.23 ± 6.41^BGb^
Mixing/1	Base of the slope	0.00	0.08 ± 0.24^Ba^	3.96 ± 1.38^BCDEa^	42.71 ± 18.88^Aa^	39.82 ± 10.47^ACa^	13.43 ± 9.74^ADa^
	Middle of the slope	0.00	0.07 ± 0.10^Aa^	4.06 ± 1.38^AFGHa^	28.23 ± 7.83^Bb^	49.25 ± 5.64^BCb^	18.39 ± 3.48^BCab^
	Top of the slope	0.00	0.01 ± 0.01^BCDEFa^	3.76 ± 2.11^AEFa^	24.86 ± 7.51^Ab^	50.15 ± 6.24^ACb^	21.21 ± 3.59^Eb^
Mixing/1.5	Base of the slope	0.00	0.55 ± 0.37^AEFa^	5.17 ± 2.78^ACa^	38.48 ± 14.27^Aa^	40.94 ± 9.57^Aa^	14.86 ± 7.66^AEa^
	Middle of the slope	0.00	0.34 ± 0.38^Aab^	5.98 ± 1.79^BDEFIa^	56.09 ± 4.68^Ab^	31.5 ± 4.19^Ab^	6.09 ± 3.07^Ab^
	Top of the slope	0.00	0.13 ± 0.22^ADGb^	2.18 ± 1.34^BCEGb^	32.69 ± 11.29^ACa^	50.49 ± 7.36^ADEc^	14.24 ± 5.35^ACFa^

Data are expressed as mean value ± standard deviation. Capitalized superscript letters mean that estimates for the same grain size class and same slope position differ significantly among different types of sand barrier (P < 0.05). Lowercase superscript letters mean that sediment grain size differs significantly for the same type of sand barrier (P < 0.05).

For samples collected from around sand barriers, sediment consisted primarily of fine and medium sand. Fine sand proportions for Straw/1, Straw/1.5, PLA/1, PLA/1.5, Mixed/1 and Mixed/1.5 were 36.75–47.42%, 29.15–49.71%, 46.22–47.80%, 12.3–39.75%, 24.86–42.71% and 32.69–56.09%, respectively. Medium sand proportions were 35.1–45.2%, 33.52–47.27%, 33.05–40.51%, 40.7–59.2%, 39.82–50.15% and 31.5–50.49%. Samples showed lower proportions of coarse sand and higher proportions of very fine sand relative to proportions observed in samples from the bare sand dune area.

Proportions of fine and medium sand measured from the base of slope of sand barrier areas exceeded those measured from bare dune samples. Proportions of fine sand measured from middle slope areas of Straw/1, Straw/1.5, PLA/1 and Mixed/1.5 barrier types varied relative to those measured from middle slope areas of bare sand dunes. Middle slope samples from PLA/1.5 and Mixed/1 showed proportions of fine sand similar to those measured from the middle slope of the bare dunes. LSD-T tests were used to analyze grain size variation among different positions along the slope for different types of sand barrier. Coarse sand proportions in samples from the middle slope of PLA/1.5 differed significantly from proportions measured from middle slope samples of bare dunes. Fine sand proportions in base of slope samples from PLA/1.5 differed from those measured from the base of slope bare dunes sample. Fine sand proportions did not differ significantly among other types of sand barriers relative to bare dunes. Straw/1, Straw/1.5 and PLA/1 samples differed significantly from bare dune samples. For Straw/1, the content of very fine sand in middle and top of the slope samples did not differ significantly from the base of slope samples but very fine sand proportions differed significantly between the middle and top of slope samples. Fine and coarse sand components did not differ according to position along the slope. Silty and very fine sand components measured from Straw/1.5 samples did not vary according to different positions along the slope. Silty, fine and coarse sand proportions in PLA/1 samples did not differ according to different position along the slope. Very fine, fine, medium and coarse sand components of PLA/1.5 samples did not differ for different positions along the slope. Silty and very fine sand proportions in Mixed/1 samples from the top of the slope differed significantly from proportions measured from other slope positions. Fine, medium and coarse sand proportions in Mixed/1 samples from the base of the slope differed significantly relative to proportions measured from other slope positions.

### Sediment grain size parameters

**Figure 6:**
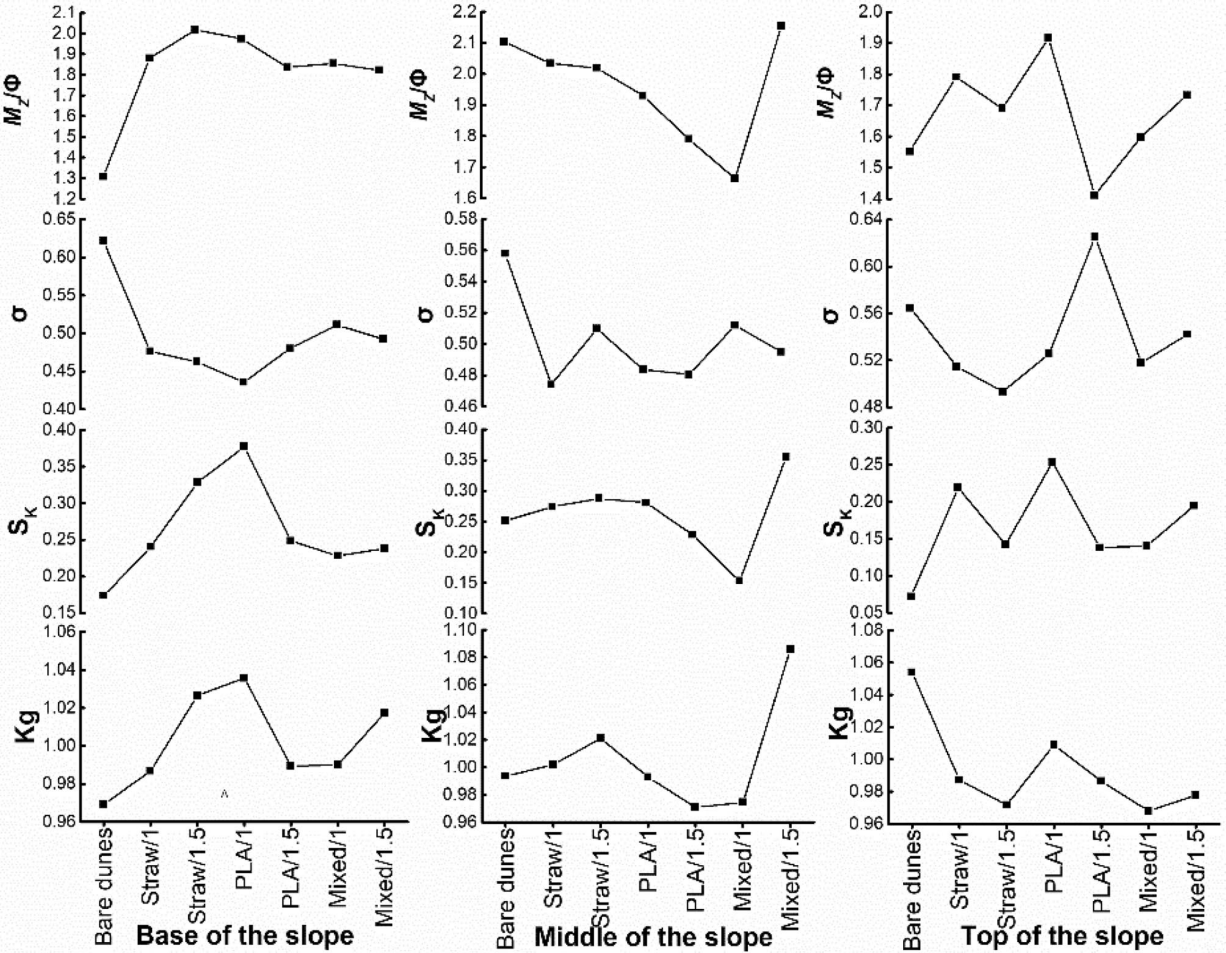
Sediment grain size parameters.

Overall, mean grain size estimates indicate coarser sediment in samples from the six types of sand barriers relative to that observed in samples from bare dune samples (Fig. 6). Mean grain size estimates for surface sediments ranged from 1.31${\Phi }$ to 2.30${\Phi }$ along bare dunes with a mean value of 1.65${\Phi }$. This indicates a predominance of medium sand. After sand barrier installation, grain size estimates for Straw/1 samples ranged from 1.79 ${\Phi }$ to 2.03 ${\Phi }$ with a mean value of 1.90 ${\Phi }$. This indicates higher proportions of coarse material relative to the bare dunes. For Straw/1.5 samples, grain size values ranged from 1.69 ${\Phi }$ to 2.02 ${\Phi }$ with a mean value is 1.91 ${\Phi }$. This value does not differ significantly from that measured from Straw/1 samples and also indicates a predominance of medium sand. For PLA/1 samples, the mean grain size ranged from 1.92 ${\Phi }$ to 1.97 ${\Phi }$ with a mean value of 1.94 ${\Phi }$. This indicates coarser sediment relative to that analyzed from straw barrier samples. For PLA/1.5, grain size ranged from 1.41 ${\Phi }$ to 1.79 ${\Phi }$ with a mean value of 1.68 ${\Phi }$. This value approaches that observed for bare dune areas. For Mixed/1 samples, mean grain size ranged from 1.60 ${\Phi }$ to 1.86 ${\Phi }$ with a mean value of 1.71 ${\Phi }$. For Mixed/1.5 samples, grain size ranged from 1.73 ${\Phi }$ to 2.15 ${\Phi }$ with a mean value of 1.90 ${\Phi }$. Mean grain size values indicate coarser sediment at the base of the slope but finer sediment in middle slope samples relative to bare dune samples.

Graphic analysis using Folk-Ward criteria gave sediment sorting values ranging from 0.56 to 0.62 with a mean value of 0.58. This categorizes as ‘better’ sediment sorting. Sorting coefficients for Straw/1, Straw/1.5 and PLA/1 ranged from 0.47 to 0.51, from 0.46 to 0.51 and from 0.44 to 0.53, respectively. Mean sorting coefficients for Straw/1, Straw/1.5 and PLA/1 were 0.49, 0.49 and 0.48, respectively. Lower values indicate improved sediment sorting which categorized as ‘good’. Sorting coefficients for PLA/1.5, Mixed/1 and Mixed/1.5 ranged from 0.48 to 0.63, from 0.51 to 0.52 and from 0.49 to 0.54, respectively. PLA/1.5, Mixed/1 and Mixed/1.5 mean values were 0.53, 0.51 and 0.51, respectively, which categorize as ‘better’. Samples from the top of the slope for Straw/1, PLA/1, PLA/1.5, Mixed/1 and Mixed/1.5 and from the middle of the slope for Straw/1.5 showed better sorting. Sorting coefficients measured in samples from the base and middle of the slope for the same type of sand barrier did not differ significantly and categorized as ‘good’.

Sediment samples from bare dune areas gave skewness values ranging from 0.07 to 0.25 with a mean value of 0.17, which categorizes as ‘fine skewed’. For Straw/1, Straw/1.5, PLA/1.5, Mixed/1 and Mixed/1.5 samples, the skewness ranged from 0.22 to 0.27, 0.14 to 0.33, 0.14 to 0.25, 0.14 to 0.23 and 0.19 to 0.36, respectively. Straw/1, Straw/1.5, PLA/1.5, Mixed/1 and Mixed/1.5 samples gave respective mean values of 0.17, 0.25, 0.21, 0.17 and 0.26. These categorize as ‘fine skewed’ and indicate coarser sediment relative to that analyzed from bare dune areas. Samples from PLA/1 gave skewness values ranging from 0.25 to 0.38 with a mean value of 0.30. These values approach ‘very fine skewed’ and exceed those measured from samples associated with other types of sand barriers. They indicate PLA/1 hosts the coarsest sediment distributions observed. Trends in skewness resemble those observed among mean grain size values for different types of sand barriers and different positions along the slope.

For samples from the bare dune area, kurtosis values ranged from 0.9693 to 1.0538 and gave a mean value of 1.0055. The frequency distribution’s leptokurtic level categorized as mesokurtic. For Straw/1, PLA/1.5 and Mixed/1 samples, kurtosis ranged from 0.9868 to 1.0020, 0.9709 to 0.9894 and 0.9678 to 0.9745, respectively. Straw/1, PLA/1.5 and Mixed/1 samples gave mean kurtosis values of 0.9920, 0.9822 and 0.775, respectively. These values were much lower than those measured from the bare dune area indicating higher concentration of sediment grain size classes than that observed from bare dune samples. Kurtosis values for Straw/1.5, PLA/1 and Mixed/1.5 samples ranged from 0.9714 to 1.0212, 0.9929 to 1.0089 and 0.9777 to 1.0172, respectively. Straw/1, PLA/1.5 and Mixed/1 samples gave respective mean kurtosis values of 1.0064, 1.0125 and 1.0269. These values indicate a mesokurtic frequency distribution. Higher kurtosis values for Straw/1, PLA/1.5 and Mixed/1 samples indicate a greater degree of grain size dispersion than that measured from bare dunes. Kurtosis values from Straw/1.5, Mixed/1 and Mixed/1.5 samples indicate greater concentration of sediment grain size classes at the base of the slope relative to the middle and top of the slope. PLA/1 and PLA/1.5 show greater concentration of sediment grain size classes at the middle of the slope relative to that measured from the base and top of the slope.

### Frequency distribution curves

**Figure 7:**
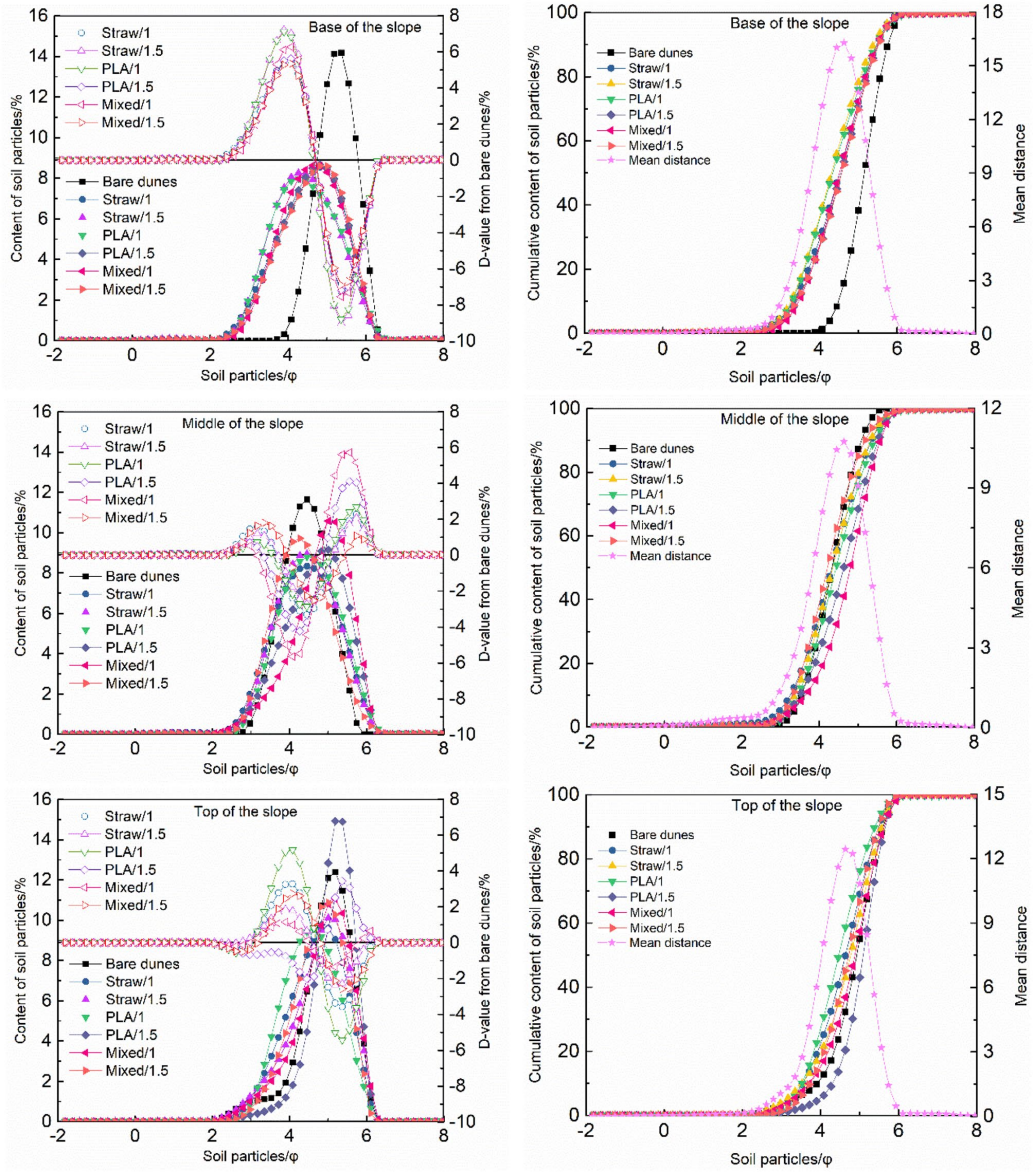
Frequency distribution curves for different samples.

Samples from the base and middle slopes of both the bare dune and sand barrier areas gave sediment frequency distribution curves consisting of a single peak. The peak broadens for all samples relative to samples from the base of the slope of bare dune areas. Sediment grain size ranges from 3.90 ${\Phi }$ to 6.30 ${\Phi }$ for bare dune samples to 2.68 ${\Phi }$ to 4.98 ${\Phi }$ for sand barrier samples. The peak value shifts from 5.19 ${\Phi }$ for bare dune samples to 8.33 ${\Phi }$ for sand barrier samples. The sand barrier samples gave sediment grain size values ranging from 2.98 ${\Phi }$ to 4.45 ${\Phi }$ whose frequency distribution curve deviated from a normal distribution. Cumulative frequency curves become very gradual and reach the top of the cumulative curve in advance. PLA/1 and Straw/1.5 samples gave broader sediment grain size distribution ranges relative to others types of sand barrier. The peak value is slightly low and slightly in advanced (Fig. 7). Frequency distribution curves for both bare dune and sand barrier samples indicate sediment consists primarily of medium sand. Sediment samples from sand barrier areas show coarsening trends relative to bare dune samples. Middle slope samples from sand barrier areas show broadened distributions and lower peak values. The peak value reached in advanced relative to the bare dunes and the value became from 4.45 ${\Phi }$ to 4.27 ${\Phi }$, the peak value at PLA/1.5 and Mixed/1 was delayed relative the bare dunes, and the value became from 4.45 ${\Phi }$ to 5.01 ${\Phi }$. Average particle size measured from Mixed/1.5 samples resembled those measured from bare dune samples. Average particle size from sand barrier samples indicated higher fine grained fractions relative to bare dune samples. Cumulative curves for sand barrier samples ranged from 2.06 ${\Phi }$ and 4.09 ${\Phi }$. This range exceeds that observed from bare dune samples. The cumulative frequency curve for sediment grain size becomes very slow, and reach the top of the cumulative curve delay. Frequency distribution curves for top of slope samples from bare dune areas vary. PLA/1.5 samples show relatively narrower distributions. Samples associated with other types of sand barrier show broader distributions. PLA/1.5 samples gives greater peak values relative to the bare dunes samples. Samples from both PLA/1.5 and bare dunes both gave a sediment grain size value of 5.19 ${\Phi }$. Peak values from samples from other types of sand barrier were low and appear in advanced relative to the bare dunes. Average particle size values for PLA/1.5 samples indicated higher proportions of fine grained material relative to bare dune samples. Straw/1, Straw/1.5 and Mixed/1.5 samples however have larger (coarser) average particle size values relative to bare dune samples. PLA/1.5 samples gave steeply sloping cumulative frequency curves.

## Discussion

Grain-size distributions of sand dunes can help constrain aeolian sedimentary processes^23,24^. As mechanisms for mitigating aeolian erosion, mechanical sand barriers can influence sediment grain size distributions. They may specifically increase the proportion of moderately erodible particles on the surface of sand dunes.

### Sand barrier sediment grain size distributions

Sand barriers appear to reduce aeolian erosion through increased surface roughness and by creating a higher wind velocity threshold for sand transport^25^. Wind tunnel experiments by Zhong et al. simulated sand control effects of a plant-based sand barrier. These showed a decline in sand accumulation with increased vegetation cover. Aeolian sand transport decreased with the increase in vegetation height^26^. Dong et al. reported an unchanged wind velocity field following installation of a sand barrier having different specifications. Areas around the sand barrier however did disrupt wind flow. Areas in front of and inside the barrier showed a reduction in wind velocity. An acceleration zone formed within the barrier decreased or prevented sand accumulation within the barrier^27^. A shift in wind velocity can influence sediment grain size distributions in sediment^28,29^. The mean value of average sediment particle size inside the sand barrier will increase with time while sediment sorting decreases. However, skewness and kurtosis do not show significant variation and sand transport occurs primarily through saltation inside the barrier. Saltation is the primary driving force by which sand accumulates to bury the barrier^18^. Xu et al. simulated wind‐sand flow around barriers using 3D hybrid Reynolds‐averaged Navier‐Stokes large eddy numerical and Lagrangian particle tracing methods to determine characteristics of turbulent flow, particle motion and erosion of barriers. The results show that the vast majority of particles fall into barrier cells from middle and posterior positions during aeolian transport. Deposition results from gravity and subsidence flow indicating that barriers effectively reduce sand transport. Turbulent flow within barriers assumes a relatively high instantaneous pulse velocity resulting in remobilization of sediment within barriers. The mean flow field within barriers consists of a streamwise vortex spanning the barrier grid cells. Two additional vortices form behind barrier cells. Together, vortices plaster particles against the front and side walls of barrier cells. Barrier grid cells develop a central depression with sediment wedges formed along the sides^30^.

The present study compared grain-size parameters in sediment collected from six types of mechanical sand barriers. Samples collected from the Straw/1 sand barrier consisted primarily of medium sand with average sorting of 0.49 (‘good’), a skewness of 0.14–0.23, (fine skewed) and a mean kurtosis value of 0.9920. Relative to samples associated with other types of sand barrier, Straw/1 samples exhibited relatively concentrated grain size distributions. Material and construction costs for the 1 × 1 m straw sand barrier grid are relatively low given the efficiency with which the barrier arrests sand transport. This type of sand barrier not only induces changes in the surface flow field but also enhances deposition of coarse sand. As such, Straw /1 represents the most effective type of sand barrier analyzed under present conditions.

### Effect of mechanical sand barrier on the range of eroded particles

**Figure 8:**
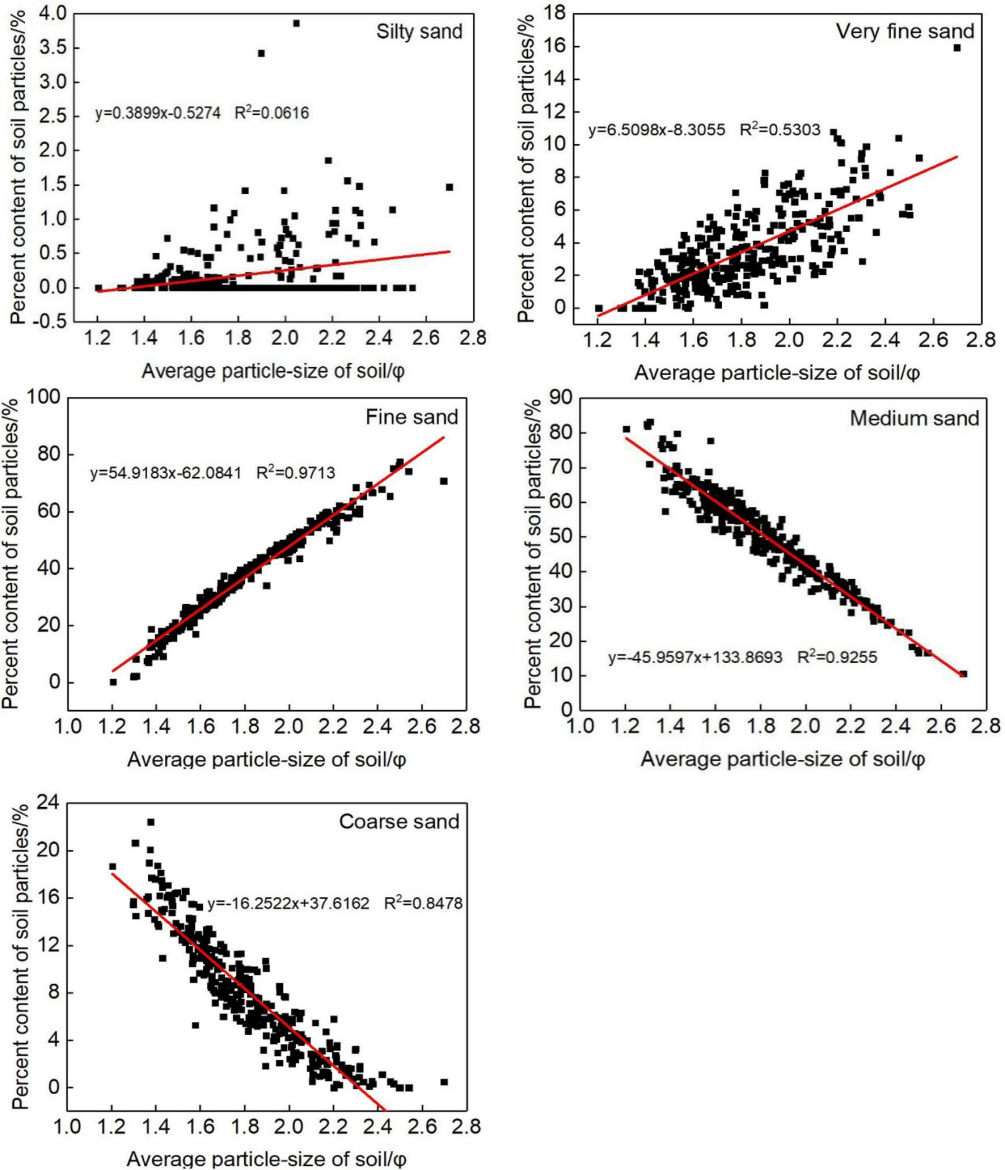
Correlations of sediment content with the average grain size of sediments around sand barriers.

Mechanical sand barriers reduce erosion and transport by increasing surface roughness and changing the direction, speed and structure of wind-sand flow^28,29,31^. Sediment grain size distributions indicate disrupted wind-sand flow patterns^32^. Cumulative frequency distributions document differences in grain size distributions between sample sites that are consistent with variation in aeolian processes^33^. Cumulative frequency distributions show sediment grain size ranges of 2.9771Φ-5.7408Φ (Fig. 8). This indicates sediment within the erosional field for aeolian processes. In terms of aeolian processes, saltation is the primary transport process. Very fine, fine and medium sand range in particle size from 1Φ to 4.3219Φ, a range susceptible to saltation. Coarse sediment having a particle size of less than 1Φ is primarily transported through creep^8,11^. Dong found that very fine, fine and medium sand is susceptible to erosion which affects sediment grain sizes between 1.3129Φ and 3.7370Φ. Coarse sediment with a grain size range of 0.5164Φ to 1.3219Φ is not susceptible to aeolian erosion. This study showed that the range of particles experiencing erosion, transport and deposition is consistent with that observed from previous studies. Eroded sediment consists primarily of very fine and fine sand, a grain size range strongly influenced by aeolian processes.

As an index of sediment grain size, average particle size can be strongly influenced by wind erosion^13^. We performed linear regression analysis of sediment mean particle size and grain size percentages for different barrier types in order to determine grain size parameters that covary with particle size distributions. The mean particle size correlated positively with very fine and fine sand (R = 0.7292, 0.9856, P < 0.05). Mean particle size correlated negatively with medium and coarse sand (R = − 0.9621, − 0.9210, P < 0.05). Analysis found poor linear fit between mean particle size and silt fraction (R = 0.2538, P < 0.05). Comprehensive analysis indicated aeolian erosion affecting very fine sand and fine sand, two particles size classes that appear in relatively high abundance in sand barrier samples.

### Effectiveness of mechanical sand barriers

Sand barriers can locally influence aeolian processes. The blocking effects of the sand barrier apparently increase proportions of medium and coarse sand while fine sand content decreased. Samples associated with sand barriers show poorer sediment sorting^14^. Previous studies have shown sand barriers reduce wind velocity^34,35^ which in turn lowers the sediment transport capacity and increases the likelihood of deposition. Wind-sand flow is supersaturated when it encounters the sand barrier. Decreased flow at base of the slope leads to deposition of medium to coarse sand^36–39^. The Straw/1.5 and PLA/1 areas show visible hollowing out relative to Straw/1, PLA/1.5, Mixed/1 and Mixed/1.5 areas. Accumulation of sediment around the sand barrier vertically raises wind-sand flow along the slope causing further deposition of coarse grained material. The wind-sand flow continues in an unsaturated state and continues to erode fine sand at the top of the slope.

The PLA sand barrier is a soft sand barrier that consists mostly of local sand fill which gives the barrier greater mass. This mass serves to stabilize the barrier and confers a shear resistance to horizontal wind force ^40,41^. The smaller the size of the sand barrier grid, the higher the density of grid surface and the greater its stability. PLA barriers thus last longer than straw barriers^40^.

While the PLA barrier material is natural and biodegradable, and thus carries a lower cost for the environment, its up-front costs exceed those of straw barriers. PLA barriers require considerable installation time and produce a loose sediment areas prone to sand accumulation. The PLA barrier compared poorly with the straw barrier in other ways as well. Due to its surface installation, the PLA barrier can be moved to address local sediment accumulation or erosion. Wind can sometimes dislodge the barriers and PLA barriers are easily deformed, both which lessens their protective effects. Labor and other resources are necessary to maintain the protective effects^42^. The Straw/1 grid gave better protection than other types of sand barriers. This sand barrier is relatively stable, with low maintenance cost and offers relatively strong erosion control. The Straw/1.5 and Mixed/1.5 barrier types follow in terms of protective benefits.

## Conclusions

1. Analysis of sediment characteristics from six types of mechanical sand barrier showed that sediment consisted primarily of fine and medium sand. Sediment grain sizes for protected areas ranged from 1.41Φ to 2.03Φ and generally hosted higher proportions of coarser sediment relative to bare dune areas used as a control. Sediment from the barrier areas exhibited good sorting, skewness values that categorized as ‘fine skewed’ and mesokurtic kurtosis. Sediment collected around the sand barriers showed lower proportions of fine sand and higher proportions of coarse sand than that measured from around bare dune areas. Samples from barrier areas showed a wider frequency distribution and cumulative frequency curves became more gradual indicating coarser sediment.
2. Sediment collected from bare dunes (used as a control) consisted primarily of fine and medium sand with a relatively low coarse sand component. Statistical analysis showed that fine and medium sand from the base of the slope for barrier areas differed significantly from analogous samples collected from bare dune areas. Variation in sediment grain size distributions indicates that the different barriers analyzed blocked wind-sand flow through different mechanisms.
3. Barriers cause coarse and medium sand to deposit along the base and middle of the slope. The top of the slope experiences erosion. Comparison of installation costs and protective effects of different barriers suggest that straw barriers installed in a 1 × 1 m grid (Straw/1) offer the greatest, long term protective benefits.
